# Early childhood SARS experience leads to long-lasting impacts on adulthood mental health in China

**DOI:** 10.1038/s41598-023-49970-w

**Published:** 2023-12-19

**Authors:** Ye Yuan, Litian Chen, Chao Yang, Tingting Xie

**Affiliations:** 1 School of Economics, Peking University, No. 5 Yiheyuan Road, Haidian District, Beijing, 100871 China; 2 Institute for Global Health and Development, Peking University, Beijing, China; 3 Renal Division, Department of Medicine, Peking University First Hospital, Peking University Institute of Nephrology, Beijing, China; 4 Research Units of Diagnosis and Treatment of Immune-Mediated Kidney Diseases, Chinese Academy of Medical Sciences, Beijing, China; 5 School of Economics, Central University of Finance and Economics, Beijing, China

**Keywords:** Human behaviour, Epidemiology

## Abstract

The association between pandemic experience and immediate mental health risks, such as depression, is well-documented, yet the long-term effects remain unclear. This study examines the impact of early childhood exposure to the 2003 SARS pandemic on adulthood mental health after 17 years in China, using data from the 2020 China Family Panel Studies (CFPS). The analysis included 6289 participants, aged 3 to 30 years during the SARS outbreak, with an average age of 35.3 years at the time of survey. Adulthood mental health was assessed using Center for Epidemiologic Studies Depression Scale (CESD) and an indicator of clinical depression. The severity of local SARS outbreaks was assessed by cumulative cases per 10,000 population. Results show that each additional case per 10,000 population was linked to a 1.617-fold (95% confidence interval (CI): 1.425–1.836) increase in odds of depression after 17 years for younger children (aged 3–12 years in 2003) relative to older cohorts (aged 13-30). This risk was higher in children from rural areas (adjusted odds ratio (aOR) 3.64; 95% CI 2.92–4.55), with poor physical health (1.98; 1.59–2.48), and from low-income families (2.87; 2.03–4.05). The childhood pandemic experience elevated the probability of developing depression-prone personality traits, which contributes to the enduring impact of childhood pandemic experiences on adulthood mental health. These findings highlight the long-lasting psychological impact of early-childhood pandemic exposure, underscoring the need for targeted interventions to mitigate its effects on the younger generation and emphasizing the importance of monitoring long-term mental health and personality development in children post-pandemics, particularly in light of COVID-19.

## Introduction

Amid the pandemic of Coronavirus disease 2019 (COVID-19), growing evidence shows that the pandemic has led to a substantial increase in the risk of depression, anxiety, stress, and other psychological disorders in the general public—and more so for vulnerable and disadvantaged groups, especially children and the poor^1–5^. Most prior studies have assessed the contemporaneous effects of pandemics on the mental health of adults. A critical yet under-addressed question is whether a major pandemic would have long-lasting psychological impacts on children, whose mental status is particularly vulnerable to memories of traumatic experience^5,6^.

Children who were separated from their families, friends, and school during the COVID-19 crisis could experience anxiety, distress, social isolation, and an abusive environment that can have short- and long-term effects on their mental health. The existing evidence on the impact of COVID-19 pandemic control policies on the mental health of children is a subject of growing concern and research. Growing evidence has shown that children experienced a higher risk of anxiety and depressive symptoms after the onset of COVID-19 crisis^5–8^. Numerous studies have explored the adverse effects of lockdowns, social distancing measures, and school closures on the psychological well-being of children. The abrupt disruption of daily routines, prolonged social isolation, and the shift to online learning have introduced new stressors and challenges for children. Research suggests an increased prevalence of symptoms related to anxiety, depression, and other mental health issues among children during the pandemic. Limited outdoor activities, reduced social interactions, and the absence of in-person educational support contribute to heightened feelings of loneliness and frustration. Understanding the nuanced interplay between pandemic control policies and children's mental health is crucial for developing targeted interventions to support their well-being during and beyond the COVID-19 pandemic. More worryingly, while existing literature has extensively explored the short-term impact of COVID-19 experience on children’s mental well-being, the literature has shown that such adverse childhood experience could have long-lasting impact on early development of the brain^9–12^, the formation of personality traits^13,14^, and the overall psychological system^15^. Assessment of the long-term impact of pandemic experience on the mental health of children is clearly warranted.

The objective of this study is to determine the association between early childhood experience of pandemic and later-life mental health. We drew evidence from the twenty-first century’s first deadly infectious disease: severe acute respiratory syndrome (SARS), which shook the world in 2003 and prompted the World Health Organization (WHO) to declare it “a worldwide health threat”^16^. China was at the epicenter of the outbreak, with 5327 confirmed cases and 349 deaths in mainland China. The outbreak immediately caused a public health crisis: It triggered anxiety, rumormongering, and resource-hoarding across the country, which provoked a downward spiral of fear and panic. In response, the Chinese government implemented a series of pandemic-control measures, including quarantine, closure of schools and other public facilities, and various mobility restrictions. The pandemic was brought under control in late June, eight months after the first known case, and all known cases were cleared by mid-August 2003.

While the pandemic was ended within a year, its impact persisted long after. Previous studies have extensively documented elevated rates of posttraumatic stress disorder, anxiety, and depression among individuals directly involved with the disease, including the infected, quarantined individuals, health professionals, and community health workers, persisting for up to two years post-pandemic^17–20^. However, the psychological repercussions of the SARS outbreak on the general population, particularly those who experienced it during early childhood, have received limited attention. Existing research on the enduring consequences of childhood traumatic experiences suggests a lasting psychological effect. Therefore, our hypothesis posits that early childhood exposure to a local SARS outbreak is linked to lower mental health levels and an increased risk of depressive symptoms in adulthood.

Furthermore, we aim to explore the potential pathways through which a months-long exposure to the SARS pandemic during early childhood may have a lasting impact on adulthood mental health. The literature has shown that childhood adverse or traumatic experience has long-lasting impacts on the formation of children’s personality traits^13,14^. In addition, early childhood is the crucial period when the development of a child’s personality traits are most susceptible to the impact of adverse experiences^21,22^. Traumatic childhood experience may alter the formation of certain personality traits that affect adulthood mental health^23,24^. For example, personality traits such as neuroticism, extraversion, and openness are highly sensitive to the influence of early-life events and are strongly associated with adulthood risk of depression^25–28^. We posit that pandemic-induced changes in personality traits during the SARS outbreak period may act as a crucial pathway to channel the impact of childhood pandemic experiences on adulthood mental health.

## Data

### Data sources and participants

This population-based, cross-sectional study was based on the 2020 China Family Panel Studies (CFPS). The survey collects individual- and household-level information to provide high-quality data on Chinese society, economy, population, education, and health. The survey selects a nationally representative sample of the Chinese population using a stratified two-stage cluster sampling design by selecting enumeration areas and households within each enumeration area^29^. The 2020 CFPS survey was conducted between July and December 2020.

We determined our analysis sample based on two criteria. First, the sample was restricted to those aged 3 to 30 in 2003, the year of the SARS outbreak. We adopted age 3 as a lower bound because children’s ability to remember specific episodes emerges in infancy and undergoes substantial improvement in the first 2 to 3 years of life^30,31^. In addition, we have insufficient sample size for children aged below 3 in 2003. Second, we required sampled participants not to have changed their residential city since early childhood. In the survey dataset, we only have information on each participant's current residence, not their childhood residence. This requirement ensures our ability to track and measure each participant's exposure to the local SARS outbreak in their childhood city of residence. In our sample of participants who were aged 3–30 in 2003, approximately 4% had relocated since early childhood and were consequently excluded from our analysis. After both sample restrictions, our analytical sample included 6 289 participants from the 2020 CFPS.

CFPS data are collected, provided, and maintained by the Institute of Social Science Survey of Peking University. This study was conducted from February 1, 2022 to September 10, 2022. We followed the Strengthening the Reporting of Observational Studies in Epidemiology (STROBE) reporting guideline for cross-sectional studies. Because all data were obtained from public domains, this study does not constitute human participant research and does not require exemption according to the US Department of Health and Human Services.

## Variables

### Mental health

Our main measure of depression was based on the 8-item Center for Epidemiologic Studies Depression Scale (CESD). CESD was initially developed by Radloff^32^ to contain 20 items, but shorter versions, such as 11-item and 8-item versions, have been widely adopted in large population surveys based on their expediency and general clinical equivalence. Prior studies have confirmed that the 8-item CESD is a valid and reliable screening measure of clinical depression, and shows good predictive accuracy compared with the 20-item CESD^33–36^. Specifically, each participant was asked eight questions regarding how often over the past week they experienced symptoms associated with depression, such as restless sleep, poor appetite, and feeling lonely. In each question, the participant was asked to rate their frequency of having a particular mental status during the past week such as loneliness, sleep disturbance, and sadness. Answers included never, sometimes, often, and most of the times, with corresponding score as 0 to 3. Scores from all questions were summed and scaled to a standard 20-item CESD score following the survey instruction. A higher score indicates worse mental health status and a higher risk of depression.

An indicator of clinical depression was defined to be one if the participant’s scaled CESD score was higher than a cutoff value of 16^32^. As a robustness check, we also set cutoff values of 18 and 20^37^. In addition, we defined an indicator for each item of CESD if the item-specific score was equal to or exceeded its midpoint score.

It is worth noting that all mental health variables were measured after the end of COVID community transmission in China, as most pandemic restrictions were lifted and the country was moving out of the COVID pandemic.

### Personality traits

The CFPS evaluated respondents’ Big Five personality traits through a set of survey questions. We construct variables of Big Five personality traits for each respondent in our CFPS sample following standard procedures^38,39^. Survey questions on different personality traits elicit responses on different scales, such as 0 to 5 or 1 to 10. To ensure comparability, we standardized the scores for each trait within a range of 0 to 1, with higher values indicating a greater likelihood of developing a specific personality trait.

### Severity of local SARS outbreak

**Figure 1:**
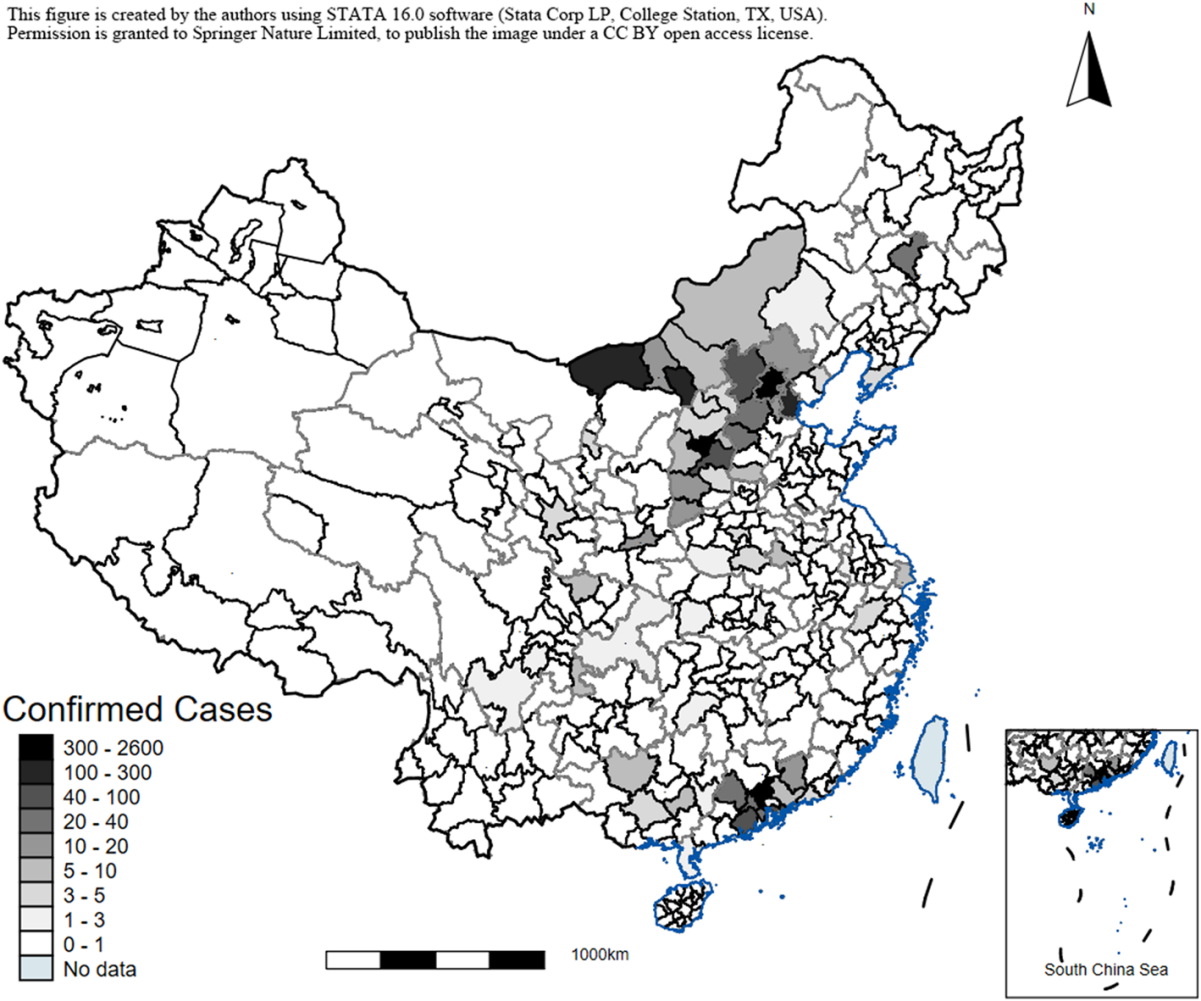
City-level Number of SARS Cases in China*. Note:* This figure shows the geographical distribution of the city-level number SARS cases in a map of China. Data from Hong Kong, Macau, and Taiwan are excluded. This figure is created by the authors using STATA 16.0 software (Stata Corp, College Station, TX, USA). Permission is granted to Springer Nature Limited to publish the image under a CC BY open access license.

We measured the severity of local SARS outbreak in a city using the number of cumulative confirmed SARS cases per 10,000 city population. We obtained this statistics from a WHO report on cumulative confirmed SARS cases in mainland China (excluding Hong Kong, Macau, and Taiwan), and obtained data on city population in 2003 from China City Statistical Yearbooks. Figure 1 plots the national distribution of this SARS severity measure in China. It shows two hot spots of confirmed cases—Beijing and Guangzhou—and a wide variation in cases in neighboring cities. We matched the severity measure of SARS to each CFPS participant based on the participant’s childhood city of residence.

We also adopt an alternative measure for the severity of local SARS outbreak using the mortality rate per 100,000 population at the province level. We obtain this statistics from China Health Statistics Yearbook 2004.

### Covariates

We included a set of demographic and socioeconomic variables that are relevant to mental health. These included gender (female vs male), marital status (married vs single, never married, widowed, or divorced), educational attainment (none, primary, secondary, and higher), employment status (full-time, part-time, unemployed), urban or rural residence, physical health index, family wealth index, and a variable representing the survey calendar month to account for seasonality. The family wealth index was a measure of participant’s self-reported family wealth status during early childhood, which measured the child’s accessibility to economic resources during their early life of development. The physical health index was measured by participant’s self-reported history of diseases and physical health status during early childhood. These variables were included in our statistical analyses as regression covariates. We estimated the effect of childhood experience of local SARS outbreak after controlling for these demographic and socioeconomic variables.

### Subgroup analyses

We assessed whether associations between childhood pandemic experience and later-life mental health differed by population subgroups. These subgroups were defined by a priori individual or family characteristics, including gender, urban vs rural residence, physical health status, and family wealth index.

## Methods

### Statistical analysis

To assess the associations between early childhood exposure to pandemic and later-life mental health outcomes, we fitted a logistic regression model to estimate the effect of a more severe SARS outbreak on the odds of later-life depression for cohorts who experienced the outbreak at early childhood (3–12 years) relative to older cohorts living in the same city. We focused on the sample aged 3 and above in 2003 due to an insufficient sample size for participants below the age of 3 in our survey data. Early childhood, defined as ages 3–12 in our study, is considered a critical period for the formation of an individual's psychological system and personality traits. Beyond age 12, a person's psychological system and the development of personality traits tend to stabilize. Consequently, we anticipate that the impact of pandemic exposure is most pronounced during this period of early childhood compared to other stages in an individual's life.

It is important to note that we measured mental health of all sample participants at their adulthood in 2020. The primary difference among participants was their age at which they experienced the SARS outbreak in 2003. Our focal group of participants was those who experienced the outbreak at their early childhood, that is, aged 3–12. We posit that this group of participants were more affected by their pandemic experience than those who experienced SARS at an older age.

We estimated the following logistic model:

1 $$Y_{ijk} = \beta_{0} + \alpha_{1} Severity_{j} \times Young_{k} + X_{ijk} ^{\prime}\pi + \theta_{j} + \lambda_{k} + \epsilon_{ijk} .$$

${Y}_{ijk}$ represents the indicator of depression in 2020 for respondent $i$ in city $j$ of age cohort $k$. We define the age cohort based on each participant’s age in 2003. In later analyses, ${Y}_{ijk}$ also measured the characteristics of personality traits, assessed in 2020 survey. $Severit{y}_{j}$ is the aforementioned severity measure of the local SARS outbreak in city* j*. $Youn{g}_{k}$ is an indicator for early childhood exposure that equals 1 if the participant was 3–12 years old during the SARS outbreak. The vector of covariates ${X}_{ijk}$ contains a set of demographic and economic variables: gender, educational attainment, marital status, employment status, urban residential status, and physical health index and family wealth index during childhood. We control for city fixed effects ${\theta }_{j}$ and birth cohort fixed effects ${\lambda }_{k}$. We cluster robust standard errors at city level to allow for correlations in outcomes across participants living in the same city. The primary coefficient of interest, ${\alpha }_{1}$, estimates the effect of a more severe SARS outbreak on the odds of later-life depression for children aged 3–12 years relative to older cohorts living in the same city. We hypothesized that the estimate of ${\alpha }_{1}$ was positive, as we expected that younger children were more vulnerable psychologically to traumatic memories of the SARS outbreak.

We hypothesize that the adverse impact of childhood exposure to SARS on later-life mental health is more pronounced when the individual experienced the local outbreak at a younger age. This impact is expected to diminish with older age at the time of exposure, and there might be no lasting effect if the person experienced the SARS outbreak as an adult. To test this hypothesis, we estimate the age-specific impact of pandemic experience on later-life mental health using logistic regression:

2 $$\begin{aligned} Y_{ijk} & = \beta_{0} + \beta_{1} Severity_{j} \times D_{3 - 5} + \beta_{2} Severity_{j} \times D_{6 - 8} + \beta_{3} Severity_{j} \\ & \quad \times D_{9 - 12} + \beta_{4} Severity_{j} \times D_{19 - 22} + \beta_{5} Severity_{j} \times D_{23 - 26} \\ & \quad + \beta_{6} Severity_{j} \times D_{27 - 30} + \gamma X^{\prime} _{ijk}+ \theta_{j} + \epsilon_{ijk} \\ \end{aligned}$$

Equation (2) presents a cross-sectional regression where all sampled participants were surveyed in 2020 but experienced the SARS outbreak at different ages. The dummy variable ${D}_{3-5}$ indicates that an individual experienced the 2003 SARS outbreak at ages 3–5 during early childhood, and other cohort dummies, ${D}_{6-8}$ to ${D}_{27-30}$, are defined similarly. The omitted reference cohort is those who experienced the SARS outbreak at ages 13–18, indicated by ${D}_{13-18}$. Each regression coefficient estimate, ${\beta }_{1}$ to ${\beta }_{6}$, reflects the relative impact compared to this reference cohort. For instance, ${\beta }_{1}$ represents the impact of experiencing the SARS outbreak on adulthood mental health if an individual experienced the outbreak at ages 3–5, relative to those who experienced the outbreak at ages 13–18. Similarly, ${\beta }_{4}$ represents the corresponding impact of experiencing the outbreak at ages 19–22 compared to those who experienced the outbreak at ages 13–18. According to our hypothesis, the estimated magnitude of ${\beta }_{1}$ to ${\beta }_{3}$ should be greater than those of ${\beta }_{4}$ to ${\beta }_{6}$.

We also conducted three sets of additional analyses. First, we performed a series of robustness analyses based on Eq. (1). This involved including additional city-level and individual-level covariates in our statistical models, testing alternative specifications of SARS severity, and assessing different definitions of depression. Second, we investigated the heterogeneous impact of early childhood pandemic experience on adulthood mental health among various subgroups of the population. We stratified the analysis by gender, rural vs urban residence, physical health status during childhood, and family wealth status during childhood. Lastly, we explored the impact of early childhood experience of the SARS outbreak on the development of an individual’s personality traits. This was achieved by estimating Eq. (1), with the measure of personality traits serving as the dependent variable.

All statistical analyses were performed using STATA 16.0 software (Stata Corp LP, College Station, TX, USA).

### Ethics approval

The China Family Panel Studies (CFPS) survey was reviewed and approved by the Institute of Social Science Survey (ISSS) of Peking University, Beijing, China. The data were released to the researchers without access to any personal identifiers. All participants and their parents provided written informed consent. All methods were performed in accordance with the relevant guidelines and regulations.

## Results

The analytic sample included 6289 survey participants aged between 3 and 30 years at the time of the SARS outbreak. The sample comprised 3 237 women (51.5%) and 3 052 men (48.5%); mean (standard deviation, SD) of age was 35.3 (7.4) years at the survey time; the average years of education was 10.4 (4.1) and about half (46.1%) had finished high school or above. More than three-fourth of participants (77.9%) were married, 86.1% were employed, and about half (52.0%) lived in the urban areas. Table 1 reports additional sociodemographic characteristics and outcomes of participants in the analytic sample. Overall, mean (SD) of severity measure of SARS outbreak in participant’s childhood city of residence was 0.07 (0.33) cumulative cases per 10,000 population. There were 53.2% of participants who experienced no local outbreak and 3.1% of participants experienced a severe outbreak of more than one case per 10,000 local population. Mean (SD) CESD-20 score was 11.1 (7.4), and 20.3% of participants were at risk of clinical depression (CESD-20 > 16).

**Table 1:** Descriptive statistics of 6 289 CFPS survey participants included in the analysis.

Variable	N	%
Demographics		
Gender		
Female	3237	51.5
Male	3052	48.5
Age, years (at 2003)		
3–12	1433	22.8
13–18	1662	26.4
19–30	2905	46.2
Educational attainment		
None	278	4.4
Primary	812	12.9
Secondary	2298	36.5
Higher	2901	46.1
Married	4901	77.9
Employed	5414	86.1
Urban residence	3272	52.0
Exposure to SARS outbreak		
0 case	3344	53.2
0–0.01 cases per 10,000	1634	26.0
0.01–1 cases per 10,000	1116	17.7
> 1 case per 10,000	195	3.1
Mental health outcomes		
CESD-20 score		
0–10	2775	44.1
10–20	2648	42.1
20–30	732	11.6
30–40	118	1.9
40–50	14	0.2
Clinical Depression (CESD-20 > 16)	1275	20.3
Personality traits score (0–1)	Mean	
Conscientiousness	0.658	
Extraversion (Positive Emotion)	0.787	
Agreeableness (Trust strangers)	0.254	
Agreeableness (Altruism)	0.729	
Neuroticism (Depression)	0.188	
Neuroticism (Anxiety)	0.250	
Neuroticism (Vulnerability)	0.069	
Openness	0.512	

**Figure 2:**
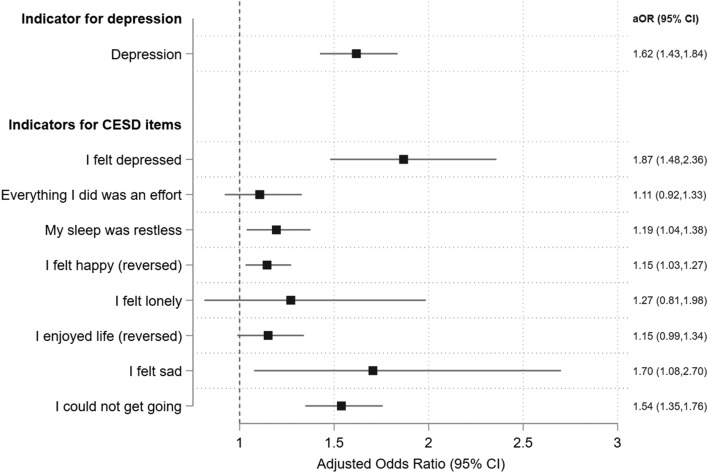
Adjusted Estimates for the Effect of Childhood SARS Severity on Odds of Later-life Depression. aOR, adjusted odds ratio; CI, confidence interval. *Note*: Indicator for depression is defined as CESD > 16. Each CESD item asked the participant about her mental status in the past week. The indicator for a specific item of CESD is defined as 1 if the participant answered “often” or “most of the times.” All models controlled for gender, marital status, employment status, years of education, urban residence, survey month fixed effects, birth year fixed effect, and city fixed effects. Standard errors account for clustering at the city level.

In logistic regression analyses (Fig. 2), we found that each one more SARS case per 10,000 population was associated with 1.62 (95% confidence interval (CI), 1.43–1.84) higher odds of depression after a lapse of 17 years for children aged 3–12 years relative to older cohorts living in the same city. Figure 2 also shows a consistent pattern of associations of childhood pandemic severity and specific items of CESD. For example, one more SARS case per 10,000 population was associated with 1.87 (1.48–2.36) higher odds of feeling depressed during the past week; 1.19 (1.04–1.38) higher odds of having restless sleep; 1.70 (1.08–2.70) higher odds of feeling sad; and 1.54 (1.35–1.76) higher odds of feeling not able to get “going” with life. In ordinary least squared analyses (eTable 1), we confirmed such strong associations (coefficient, 0.07; 95% CI 0.05–0.09) in the likelihood of depression and the overall CESD score (coefficient, 1.01; 95% CI 0.27–1.75). We also adopt the SARS mortality rate as an alternative measure of SARS severity and estimate the baseline logistic regression (eTable 2). We found consistently higher odds (adjusted odds ratio (aOR) 1.922; 95% CI 1.590–2.322) of adulthood depression associated with early childhood exposure to the SARS outbreak.

**Figure 3:**
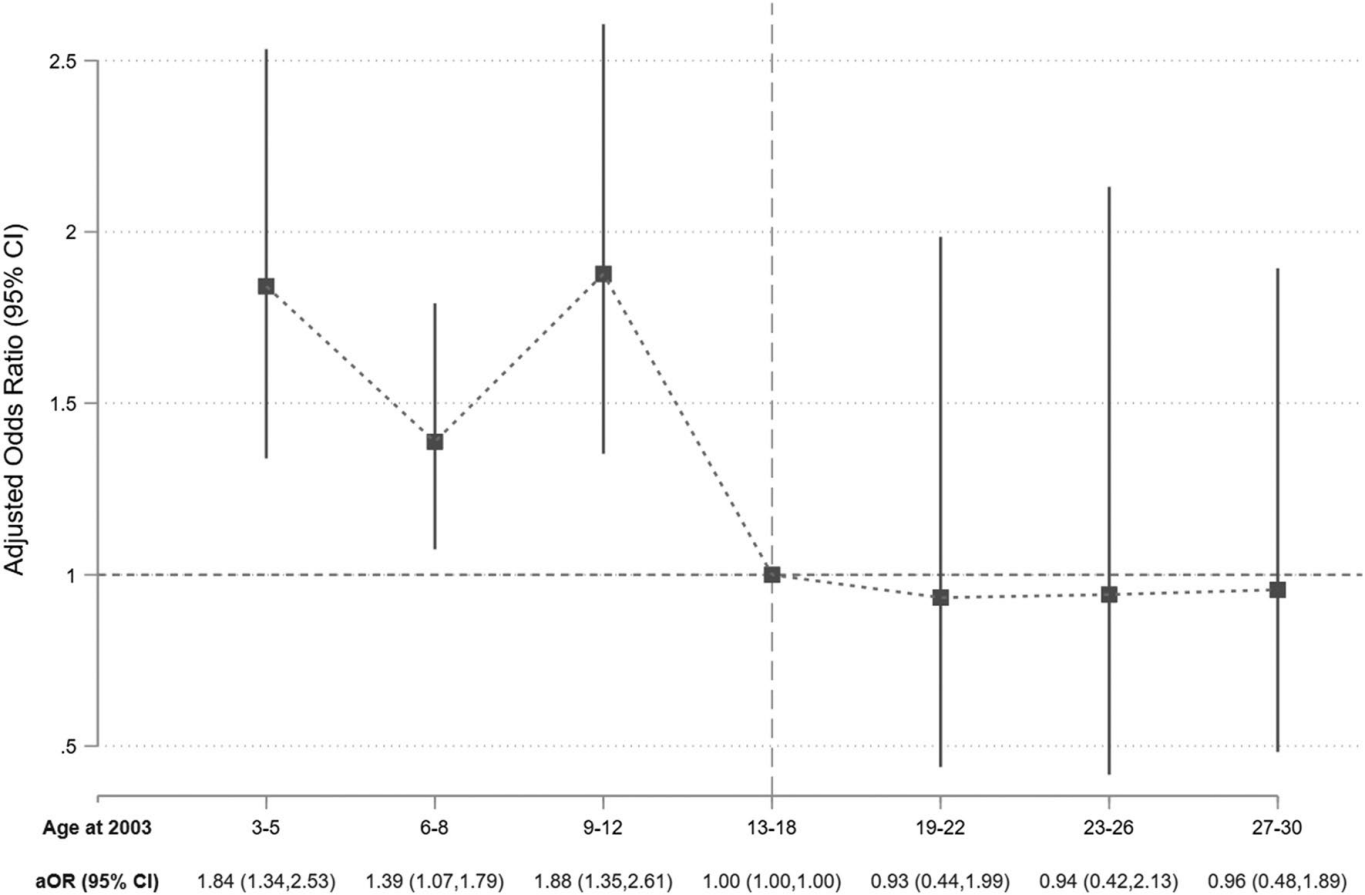
Adjusted Estimates for the Age-dependent Effects of Childhood SARS Severity on Odds of Later-life Depression. aOR, adjusted odds ratio; CI, confidence interval. *Note*: Indicator for depression is defined as CESD > 16. The model controlled for gender, marital status, employment status, years of education, urban residence, survey month fixed effects, and city fixed effects. Standard errors account for clustering at the city level.

Figure 3 plots the adjusted estimates for age-specific association of SARS severity and later-life depression. As consistent with the literature on the imprint effect of childhood traumatic experience^40–43^, the estimated association of childhood SARS severity and later-life depression were greater for children aged 3–5 years (aOR 1.84; 95% CI 1.34–2.53), 6–8 years (1.39; 1.07–1.79), and 9–12 years (1.88; 1.35–2.61), relative to the reference cohorts aged 13–18 years. In other words, participants who experienced a severe SARS outbreak at early childhood (3–12 years) exhibited greater odds of depression in later life relative to the reference cohort who experienced the outbreak at later childhood (13–18 years). In contrast, participants who experienced the outbreak at an older age (19–30 years) exhibited similar odds of depression relative to the reference cohort.

**Figure 4:**
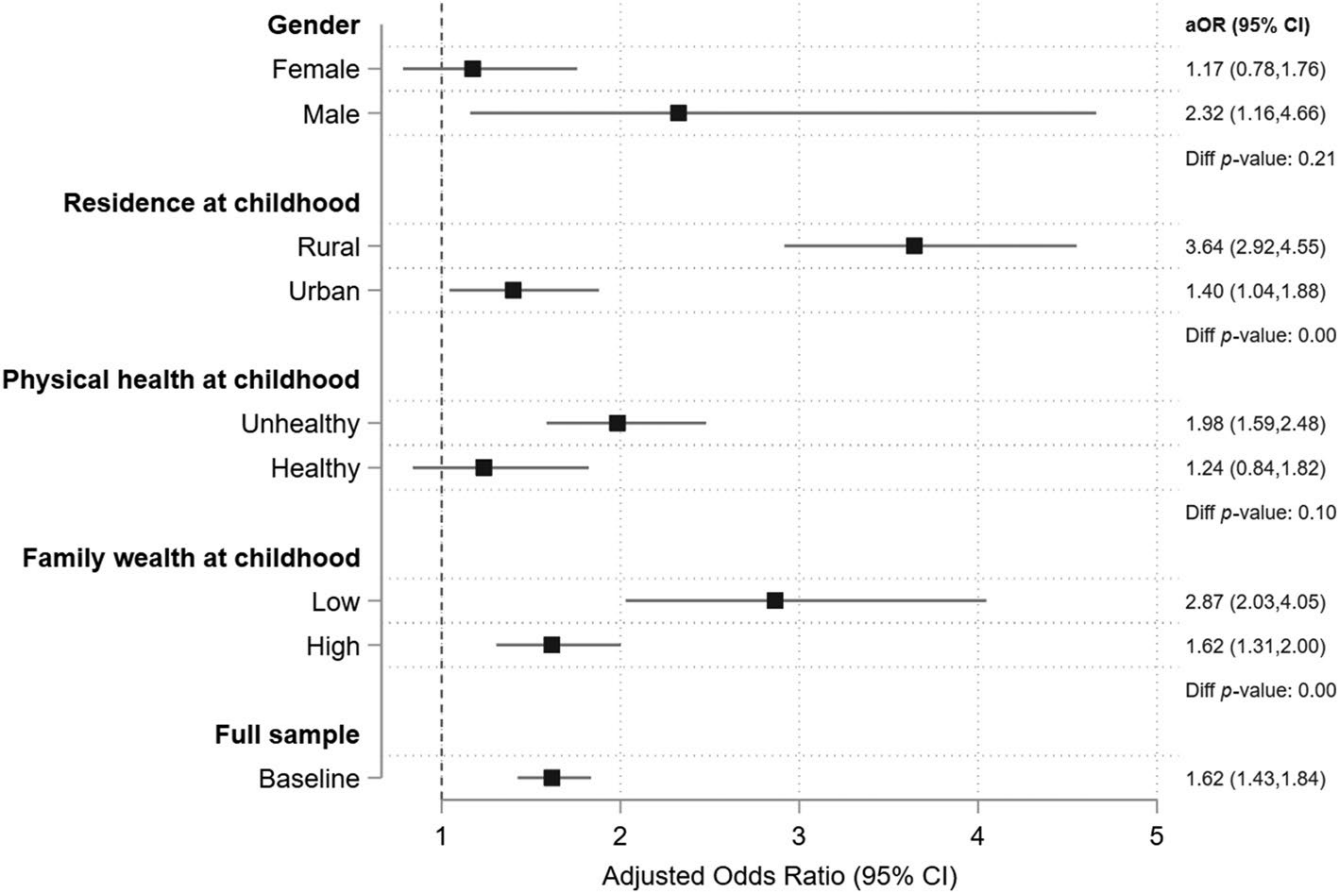
Adjusted estimates for the heterogeneous effect of childhood SARS severity on odds of later-life depression, stratified by subsamples. aOR, adjusted odds ratio; CI, confidence interval. *Note*: Indicator for depression is defined as CESD > 16. Participants are stratified by gender, rural vs urban residence during childhood, above vs below mean of physical health status during childhood, and above vs below mean of family wealth index during childhood. All models controlled for gender, marital status, employment status, years of education, urban residence, survey month fixed effects, birth year fixed effect, and city fixed effects. Standard errors account for clustering at the city level.

Figure 4 presents the adjusted estimates for the association between local SARS severity and later-life onset of depression, stratified by gender, rural vs urban residence, physical health status during childhood, and family wealth status during childhood. We found that the association of childhood SARS severity and later-life depression was greater for children from rural areas (aOR, 3.64; 95% CI 2.92–4.55), with low physical health (1.98; 1.59–2.48), and from low-income families (2.87; 2.03–4.05). We found a stronger association for males (2.32; 1.16–4.66), but the difference was not statistically significant (*p*-value of difference, 0.21).

We conducted a series of sensitivity analyses in the supplement. First, in eTable 3, we controlled a set of measures for city-level public investment in health and education during each participant’s childhood, including fiscal expenditure per capita, the population share of hospitals, hospital beds, and doctors; and the population share of primary schools. Second, we incorporated key determinants of adult mental health, such as gender, marital status, education, employment, urban or rural residence, physical health, family wealth, and the survey month, sequentially into the statistical model in Eq. (1). The estimation results, presented in eTable 4, consistently align with our baseline findings in Fig. 1, after accounting for these determinants. Third, in eTable 5, we expanded our analytical sample to include those aged 0–2 years and 31–35 years in 2003. Fourth, we defined the depression dummy with alternative cutoff score of 18 and 20 (eTable 6). Results from these analyses are consistent with the baseline finding. Lastly, we conduct two permutation tests by randomly assigning the SARS severity measures across cities (eFigure 1) and randomly assigning the age of exposure across participants (eFigure 2). Both tests showed no association between the randomly assigned childhood SARS exposure and actual later-life odds of depression.

**Figure 5:**
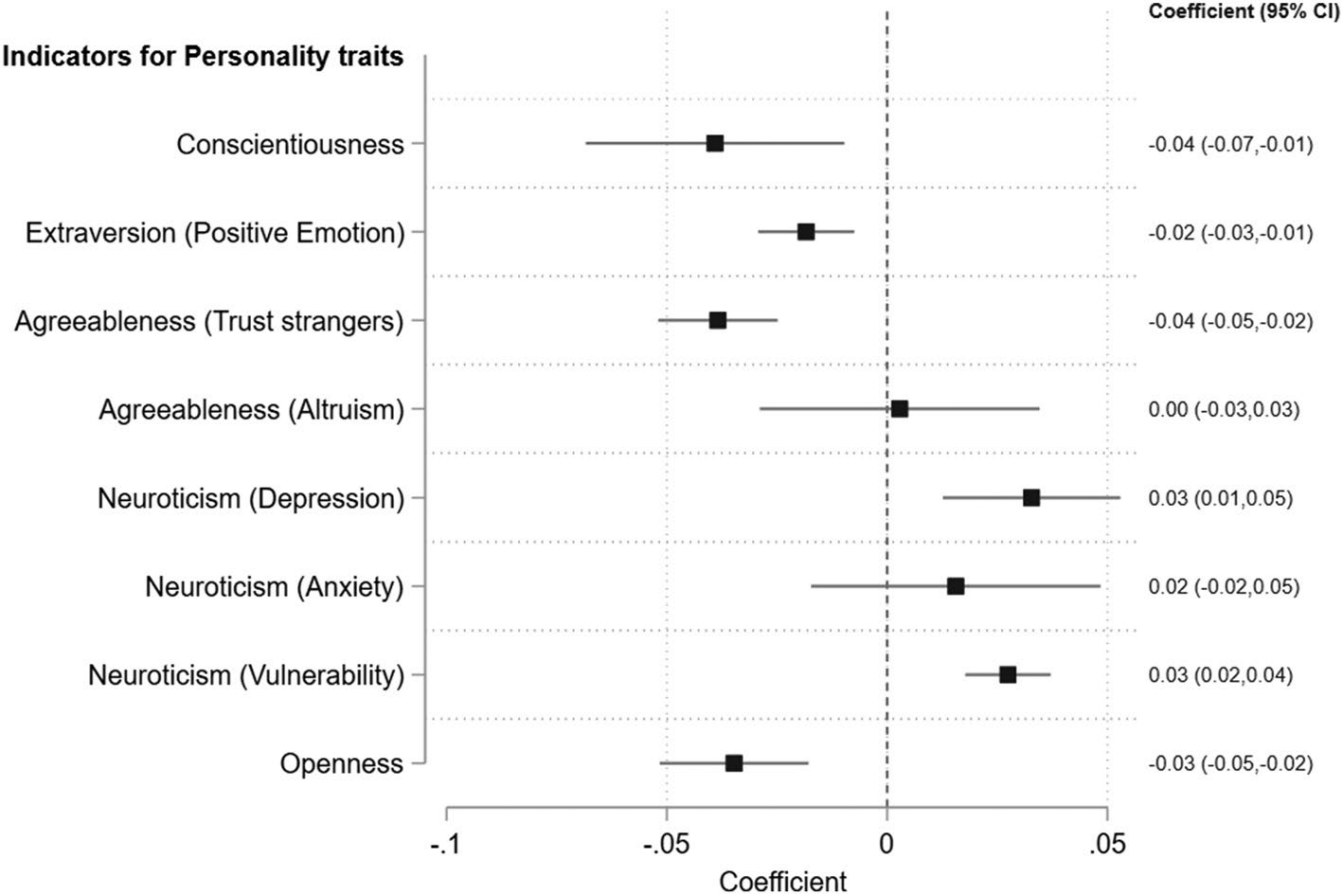
Adjusted Estimates for the Effects of Childhood SARS Severity on development of Big Five personality traits. CI, confidence interval. *Note*: Each original response to the personality trait survey questions falls within the range of 0 to 5 or 0 to 10. To ensure comparability across traits, we standardize each variable to a common scale of 0 to 1. The estimated coefficients in this figure represent the estimated change in the likelihood of developing a certain personality trait. The model controlled for gender, marital status, employment status, years of education, urban residence, survey month fixed effects, and city fixed effects. Standard errors account for clustering at the city level.

Finally, we explore whether changes in personality traits induced by the pandemic could serve as the explanatory mechanism for the enduring impact of SARS on children's later-life mental health, despite its relatively short duration. We reestimate Eq. (1) using each personality trait as a dependent variable. We find that early-life exposure to SARS significantly influenced the development of personality traits in children. In Fig. 5, we illustrate the estimated effects, along with 95% confidence intervals, of early-life SARS experience on the formation of Big Five personality traits. Specifically, early-childhood SARS experience hindered the development of conscientiousness, extraversion (positive emotions), and agreeableness (trust for strangers). Conversely, it elevated the level of neuroticism (characterized by depression, anxiety, and vulnerability) and reduced the level of openness to experience. We found no evidence of any impact of early-life SARS experience on the trait of altruism. Previous studies have consistently shown that lower levels of conscientiousness, extraversion, and openness, coupled with higher levels of neuroticism, are strongly associated with an elevated risk of depression and other mental disorders^25–28^. The findings presented in Fig. 5 indicate that the development of these depression-related personality traits serves as a crucial link between early-life exposure to a severe SARS outbreak and a higher risk of depression in adulthood.

## Discussion

This cohort difference-in-differences study based on a nationally representative survey showed that childhood SARS experience substantially elevated the risk of depression in adulthood almost two decades after the last confirmed case of the pandemic. This effect was more pronounced for the more disadvantaged children. Importantly, this long-term negative psychological impact was statistically significant only for those who experienced SARS in early childhood (aged 3 to 12), with the effect rapidly diminishing for older age cohorts. This age-dependent pattern is consistent with the imprint effect of childhood trauma and adversity on adulthood mental health.

The childhood experience of SARS pandemic meaningfully changed the formation of children’s personality traits, and especially traits associated with a higher risk of depression. Long-standing research suggests a correlation between personality traits and vulnerability to depression and other mental illnesses^44–46^. Traits like high neuroticism and low extraversion and openness are notably linked to depression risk^25–28^. Since individual responses to stress and depression are closely tied to personality traits^47^, any alterations in these traits during childhood could have lasting implications for psychological well-being^23,24^. Personality traits are generally stable in adulthood, but early childhood is a crucial period for their formation, highly susceptible to major life events^21,22^. This study presents population-based evidence from the SARS pandemic, showing how such experiences can alter children's personality trait development, particularly those traits linked to depression and mental illness. Furthermore, these personality traits are a key mechanism through which adverse childhood experiences can have enduring effects on mental health.

This paper contributes to the growing literature on the societal impact of pandemics^9–12,48–51^. Previous studies examines how large-scale pandemics like SARS, H1N1 flu, Ebola, and COVID-19 impact mental health^52–55^. These studies have focused on the short-term effects (1–2 years) of these pandemics, noting severe impacts on mental health due to the stress, fear, and isolation resulting from lockdowns, separation from loved ones, and suspension of services. However, these studies, with follow-ups of up to two years, have been unable to determine if the mental health impacts are short-lived, recovering post-pandemic, or if they persist long-term. This paper addresses this gap, exploring the extended mental health consequences of pandemics.

This paper is among the first to highlight the fact that the psychological impacts of pandemic experience on children could persist for decades. Amid accumulating evidence on the effects of the COVID-19 pandemic on risk of depression, anxiety, stress, and other psychological disorders in the general public^1–4^, most studies have focused on contemporaneous effects on the mental health of the working-aged and elderly. Our study adds critical evidence on the long-term effects of pandemic experience on children’s mental health to this literature.

We aim to raise awareness among international communities and policymakers about the potential lasting mental health consequences of COVID-19 for children. Despite prior warnings following the 2003 SARS outbreak, the world was ill-prepared for COVID-19's magnitude and severity, which exceeded SARS in mortality and economic impact (refer to the distribution of COVID-19 mortality in eFigure 3). The prolonged nature and strict measures of COVID-19, along with its unique stressors like prolonged isolation, educational disruptions, and health concerns, suggest that its psychological impact on children may surpass that of SARS. Our findings on SARS' long-term mental health effects provide a baseline for estimating the potential extended psychological impact of COVID-19 on today's children. Understanding and addressing these long-term effects is crucial for developing effective public health strategies and interventions as we recover from the pandemic.

## Strengths and limitations

This study has the advantages of using a large, nationally representative dataset and rigorous statistical methods to assess the long-term psychological impacts of a major pandemic on children’s mental health. However, several limitations should be noted. First, we measure the adversity experienced during the SARS pandemic using the number of cumulative SARS cases at city level. This measure cannot distinguish the potentially different level of adversity experienced by each individual, such as whether one was ever quarantined, any family members or friends were infected, or the level of disruption to one’s social life. Second, the number of confirmed cases may be a rough measure of the SARS-period adversity children experienced: Some cities may have implemented strict pandemic-control measures and have low rates of confirmed cases, but these pandemic-control measures might have created an abusive environment that caused children to have traumatic memories of the pandemic. Third, if available, more measures on the economic cost of mental illnesses, such as consultation fee to psychologist, medication expenditure, and days of sick leave due to mental illnesses, could be analyzed to assess the long-term economic burden of the pandemic.

## Conclusion

In conclusion, our study evaluates the impact of the first deadly pandemic in the twenty-first century (SARS) on the long-term mental health of children. Our findings indicate significant, enduring psychological harm and increased disease burden from such intense childhood pandemic experiences. We present evidence that such experiences can alter children's personality trait development, particularly those traits linked to depression and mental illness, which serves as key mechanisms through which adverse childhood experiences can have enduring effects on mental health.

Our research underscores the enduring influence of childhood exposure to a public health crisis. As we grapple with the repercussions of the COVID-19 pandemic, understanding and addressing the potential long-term effects on the mental health of today's children becomes paramount for public health strategies and interventions.

### Supplementary Information

Supplementary Information.

## Data Availability

All data analyzed in this study can be accessed through Peking University Open Research Data Platform, by creating an account and filling out a brief form describing intended analyses at https://opendata.pku.edu.cn/.
