# Supplementary material for: Early childhood SARS experience leads to long-lasting impacts on adulthood mental health in China
Source: Sci Rep. 2023 Dec 19;13:22572. doi: 10.1038/s41598-023-49970-w (PMC10730901; doi:10.1038/s41598-023-49970-w)
Supplement: Supplementary file 1 — Supplementary Information. [file 41598_2023_49970_MOESM1_ESM.docx]

**Supplementary Online Content
 Early Childhood SARS Experience Leads to Long-lasting Impacts on Adulthood Mental Health in China**

Ye Yuan, Litian Chen, Chao Yang, Tingting Xie

^*^Corresponding author: Ye Yuan ([yuanye.econ@pku.edu.cn](mailto:yuanye.econ@pku.edu.cn))

**eTable 1:** Associations Between Childhood SARS Severity on Adulthood Mental Health, Aged 3-30 Years in 2003, based on Logistic and OLS Estimation

**eTable 2**: Associations Between Childhood SARS Severity and Adulthood Depression, Aged 3-30 Years in 2003, Alternative measure of SARS severity (SARS mortality rate: Death per 100 000 population)

**eTable 3**: Associations Between Childhood SARS Severity and Adulthood Mental Health, Aged 3-30 Years in 2003, Controlling for Cohort-specific City Covariates

**eTable 4**: Associations Between Childhood SARS Severity and Adulthood Mental Health, Aged 3-30 Years in 2003, after inclusion of additional covariates

**eTable 5:** Associations Between Childhood SARS Severity and Adulthood Mental Health, Aged 0-35 Years in 2003

**eTable 6**: Associations Between Childhood SARS Severity and Adulthood Depression, Aged 3-30 Years in 2003, Alternative Cutoffs for Depression Indicator

**eTable 7**: Associations Between Childhood SARS Severity on Adulthood Mental Health, Aged 3-30 Years in 2003, based on OLS Estimation

**eTable 8**: Associations Between Childhood SARS Severity on Adulthood Mental Health, Alternative Age Ranges in 2003, Based on Logistic Estimation

**eFigure 1**: Permutation Tests on the Association of Childhood SARS Severity and Adulthood Depression, Aged 3-30 Years in 2003, Randomly Assigned SARS Severity Measure

**eFigure 2**: Permutation Tests on the Association of Childhood SARS Severity and Adulthood Depression, Aged 3-30 Years in 2003, Randomly Assigned Individual’s Age in 2003

**eFigure 3**: City-level Number of Cumulative COVID-19 Cases in China

**eTable 1: Associations Between Childhood SARS Severity on Adulthood Mental Health, Aged 3-30 Years in 2003, based on OLS Estimation**

|  | (1) | | (2) | |
| --- | --- | --- | --- | --- |
| Model | **Logistic model** | | **OLS model** | |
| Dependent Variable | **Depression (CESD-20>16))** | | **CESD-20 Score** | |
| Coefficient | $Severity\times Young$ | | $Severity\times Young$ | |
| Variable | **aOR (95% CI)** | ***P* value** | **Coef (95% CI)** | ***P* value** |
| Full sample | 1.617 (1.425-1.836) | <0.001 | 1.012 (0.273-1.751) | 0.008 |
| Stratified by gender |  |  |  |  |
| Female | 1.174 (0.784-1.758) | 0.436 | 0.538 (-0.144-1.221) | 0.121 |
| Male | 2.325 (1.160-4.660) | 0.017 | 1.188 (-0.188-2.564) | 0.090 |
| Stratified by rural/urban |  |  |  |  |
| Rural | 3.643 (2.917-4.551) | <0.001 | 4.818 (4.333-5.303) | <0.001 |
| Urban | 1.401 (1.044-1.880) | 0.025 | 0.364 (-0.214-0.942) | 0.215 |
| Stratified by physical health at childhood |  | |  | |
| Low | 1.983 (1.587-2.479) | <0.001 | 1.234 (-0.158-2.626) | 0.082 |
| High | 1.236 (0.838-1.823) | 0.284 | 0.526 (-0.516-1.568) | 0.320 |
| Stratified by family wealth status at childhood |  | |  | |
| Low | 2.865 (2.029-4.046) | <0.001 | 2.782 (0.603-4.961) | 0.013 |
| High | 1.617 (1.306-2.002) | <0.001 | 0.866 (0.374-1.357) | 0.001 |

Abbreviations: aOR, adjusted odds ratio; Coef, coefficient; OLS, ordinary least squared

All models controlled for sex, marital status, age, years of education, wealth index, urban residence, and survey month. Standard errors account for clustering at the city level.

**eTable 2: Associations Between Childhood SARS Severity and Adulthood Depression, Aged 3-30 Years in 2003, Alternative measure of SARS severity (SARS mortality rate: Death per 100 000 population)**

|  | (1) | |
| --- | --- | --- |
| Dependent Variable | Depression  (CESD-20>16)) | |
| Variable | **aOR**  **(95% CI)** | ***P* value** |
| $\boldsymbol{Severity\times Young}$ | 1.922  (1.590-2.322) | <0.001 |
| Gender (male=1) | 0.965  (0.844-1.105) | 0.607 |
| Years of education | 0.930  (0.909-0.952) | <0.001 |
| Married | 0.584  (0.473-0.720) | <0.001 |
| Employed | 0.685  (0.567-0.828) | <0.001 |
| Urban residence | 1.012  (0.852-1.203) | 0.892 |
| City fixed effects | Yes | |
| Cohort fixed effects | Yes | |
| R2 | 0.061 | |
| Observations | 6289 | |

Abbreviations: aOR, adjusted odds ratio; Coef, coefficient; OLS, ordinary least squared.

All models controlled for sex, marital status, age, years of education, wealth index, urban residence, and survey month. Standard errors account for clustering at the city level.

**eTable 3:** **Associations Between Childhood SARS Severity and Adulthood Mental Health, Aged 3-30 Years in 2003, Controlling for Cohort-specific City Covariates**

|  | (1) | | (2) | | (3) | |
| --- | --- | --- | --- | --- | --- | --- |
| Model | **Logistic** | | **OLS** | | **OLS** | |
| Dependent Variable | Depression  (CESD-20>16)) | | Depression  (CESD-20>16)) | | CESD-20 Score | |
| Variable | **aOR**  **(95% CI)** | ***P* value** | **Coef**  **(95% CI)** | ***P* value** | **Coef**  **(95% CI)** | ***P* value** |
| $\boldsymbol{Severity\times Young}$ | 1.640  (1.443-1.865) | <0.001 | 0.069  (0.051-0.087) | <0.001 | 1.062  (0.306-1.818) | 0.006 |
| Gender (male=1) | 0.965  (0.843-1.104) | 0.601 | -0.005  (-0.026-0.015) | 0.604 | -0.631  (-0.991-0.271) | 0.001 |
| Years of education | 0.931  (0.910-0.953) | <0.001 | -0.011  (-0.015- -0.008) | <0.001 | -0.209  (-0.280- -0.138) | <0.001 |
| Married | 0.586  (0.474-0.724) | <0.001 | -0.087  (-0.123- -0.051) | <0.001 | -1.955  (-2.603- -1.307) | <0.001 |
| Employed | 0.688  (0.570-0.831) | <0.001 | -0.062  (-0.094- -0.029) | <0.001 | -0.743  (-1.356- -0.130) | 0.018 |
| Urban residence | 1.010  (0.850-1.200) | 0.909 | 0.003  (-0.024-0.029) | 0.839 | 0.171  (-0.390-0.733) | 0.548 |
| City fixed effects | Yes | | Yes | | Yes | |
| Cohort fixed effects | Yes | | Yes | | Yes | |
| Survey Month  fixed effects | Yes | | Yes | | Yes | |
| Pseudo R2/R2 | 0.063 | | 0.069 | | 0.088 | |
| Observations | 6198 | | 6289 | | 6289 | |

Abbreviations: aOR, adjusted odds ratio; Coef, coefficient; OLS, ordinary least squared.

All models controlled for sex, marital status, age, years of education, wealth index, urban residence, and survey month. Standard errors account for clustering at the city level.

**eTable 4: Associations Between Childhood SARS Severity and Adulthood Mental Health, Aged 3-30 Years in 2003, after inclusion of additional covariates**

|  | (1) | | (2) | | (3) | | (4) | | (5) | | (5) | |
| --- | --- | --- | --- | --- | --- | --- | --- | --- | --- | --- | --- | --- |
| Dependent Variable | Depression  (CESD-20>16)) | | Depression  (CESD-20>16)) | | Depression  (CESD-20>16)) | | Depression  (CESD-20>16)) | | Depression  (CESD-20>16)) | | Depression  (CESD-20>16)) | |
| Variable | **aOR**  **(95% CI)** | ***P* value** | **aOR**  **(95% CI)** | ***P* value** | **aOR**  **(95% CI)** | ***P* value** | **aOR**  **(95% CI)** | ***P***  **value** | **aOR**  **(95% CI)** | ***P***  **value** | **aOR**  **(95% CI)** | ***P***  **value** |
| $\boldsymbol{Severity\times Young}$ | 1.766  (1.576-1.979) | <0.001 | 1.772  (1.579-1.989) | <0.001 | 1.686  (1.494-1.903) | <0.001 | 1.590  (1.401-1.804) | <0.001 | 1.618  (1.426-1.836) | <0.001 | 1.617  (1.425-1.836) | <0.001 |
| Gender (male=1) |  |  | 0.919  (0.811-1.041) | 0.184 | 0.966  (0.852- 1.095) | 0.585 | 0.911  (0.802- 1.034) | 0.149 | 0.965  (0.844- 1.104) | 0.603 | 0.965  (0.844- 1.105) | 0.608 |
| Years of education |  |  |  |  | 0.931  (0.910-0.952) | <0.001 | 0.928  (0.908-0.949) | <0.001 | 0.931  (0.910-0.952) | <0.001 | 0.930  (0.909-0.952) | <0.001 |
| Married |  |  |  |  |  |  | 0.589  (0.478-0.727) | <0.001 | 0.586  (0.475-0.724) | <0.001 | 0.586  (0.475-0.724) | <0.001 |
| Employed |  |  |  |  |  |  |  |  | 0.684  (0.566-0.826) | <0.001 | 0.684  (0.566-0.827) | <0.001 |
| Urban residence |  |  |  |  |  |  |  |  |  |  | 1.011  (0.851-1.202) | 0.898 |
| City fixed effects | Yes | | Yes | | Yes | | Yes | | Yes | | Yes | |
| Cohort fixed effects | Yes | | Yes | | Yes | | Yes | | Yes | | Yes | |
| Pseudo R2 | 0.046 | | 0.046 | | 0.055 | | 0.059 | | 0.061 | | 0.061 | |
| Observations | 6198 | | 6198 | | 6198 | | 6198 | | 6198 | | 6198 | |

Abbreviations: aOR, adjusted odds ratio; Coef, coefficient; OLS, ordinary least squared. Standard errors account for clustering at the city level.

**eTable 5: Associations Between Childhood SARS Severity and Adulthood Mental Health, Aged 0-35 Years in 2003**

|  | (1) | | (2) | | (3) | |
| --- | --- | --- | --- | --- | --- | --- |
| Model | **Logistic** | | **OLS** | | **OLS** | |
| Dependent Variable | Depression  (CESD-20>16)) | | Depression  (CESD-20>16)) | | CESD-20 Score | |
| Variable | **aOR**  **(95% CI)** | ***P* value** | **Coef**  **(95% CI)** | ***P* value** | **Coef**  **(95% CI)** | ***P* value** |
| $\boldsymbol{Severity\times Young}$ | 1.572  (1.362-1.815) | <0.001 | 0.064  (0.046-0.081) | <0.001 | 0.996  (0.287-1.704) | 0.006 |
| Gender (male=1) | 0.880  (0.783-0.988) | 0.030 | -0.020  (-0.038- -0.002) | 0.031 | -0.873  (-1.209- -0.538) | <0.001 |
| Years of education | 0.935  (0.918-0.951) | <0.001 | -0.011  (-0.014- -0.008) | <0.001 | -0.240  (-0.296- -0.183) | <0.001 |
| Married | 0.538  (0.449-0.644) | <0.001 | -0.106  (-0.138- -0.074) | <0.001 | -2.377  (-2.982- -1.772) | <0.001 |
| Employed | 0.684  (0.582-0.805) | <0.001 | -0.065  (-0.094- -0.035) | <0.001 | -0.769  (-1.329- -0.210) | 0.007 |
| Urban residence | 1.015  (0.873-1.180) | 0.848 | 0.003  (-0.021-0.027) | 0.800 | 0.298  (-0.269-0.864) | 0.301 |
| City fixed effects | Yes | | Yes | | Yes | |
| Cohort fixed effects | Yes | | Yes | | Yes | |
| Survey Month  fixed effects | Yes | | Yes | | Yes | |
| Pseudo R2/R2 | 0.062 | | 0.069 | | 0.090 | |

Abbreviations: aOR, adjusted odds ratio; Coef, coefficient; OLS, ordinary least squared.

All models controlled for sex, marital status, age, years of education, wealth index, urban residence, and survey month. Standard errors account for clustering at the city level.

**eTable 6: Associations Between Childhood SARS Severity and Adulthood Depression, Aged 3-30 Years in 2003, Alternative Cutoffs for Depression Indicator**

|  | (1) | | (2) | | (3) | | (4) | |
| --- | --- | --- | --- | --- | --- | --- | --- | --- |
| Model | **Logistic** | | **OLS** | | **Logistic** | | **OLS** | |
| Dependent Variable | Depression  (CESD-20>18)) | | Depression  (CESD-20>18)) | | Depression  (CESD-20>20)) | | Depression  (CESD-20>20)) | |
| Variable | **aOR**  **(95% CI)** | ***P* value** | **Coef**  **(95% CI)** | ***P* value** | **aOR**  **(95% CI)** | ***P* value** | **Coef**  **(95% CI)** | ***P* value** |
| $\boldsymbol{Severity\times Young}$ | 1.407  (0.996-1.988) | 0.053 | 0.036  (-0.005-0.076) | 0.086 | 1.538  (1.136-2.082) | 0.005 | 0.023  (0.000-0.045) | 0.049 |
| Gender (male=1) | 0.906  (0.776-1.058) | 0.213 | -0.011  (-0.028- -0.006) | 0.218 | 0.904  (0.760- 1.074) | 0.250 | -0.008  (-0.022- -0.006) | 0.244 |
| Years of education | 0.926  (0.902-0.951) | <0.001 | -0.009  (-0.013- -0.006) | <0.001 | 0.927  (0.900-0.955) | <0.001 | -0.007  (-0.009- -0.004) | <0.001 |
| Married | 0.563  (0.443-0.716) | <0.001 | -0.070  (-0.100- -0.039) | <0.001 | 0.626  (0.472-0.828) | 0.001 | -0.041  (-0.067- -0.015) | 0.002 |
| Employed | 0.674  (0.537-0.847) | 0.001 | -0.049  (-0.080- -0.018) | 0.002 | 0.697  (0.541-0.898) | 0.005 | -0.032  (-0.057- -0.007) | 0.012 |
| Urban residence | 1.076  (0.876-1.320) | 0.485 | 0.010  (-0.014-0.033) | 0.416 | 1.162  (0.900-1.501) | 0.250 | 0.013  (-0.007-0.033) | 0.200 |
| City fixed effects | Yes | | Yes | | Yes | | Yes | |
| Cohort fixed effects | Yes | | Yes | | Yes | | Yes | |
| Survey Month  fixed effects | Yes | | Yes | | Yes | | Yes | |
| Pseudo R2/R2 | 0.070 | | 0.066 | | 0.072 | | 0.054 | |

Abbreviations: aOR, adjusted odds ratio; Coef, coefficient; OLS, ordinary least squared.

All models controlled for sex, marital status, age, years of education, wealth index, urban residence, and survey month. Standard errors account for clustering at the city level.

**eTable 7: Associations Between Childhood SARS Severity on Adulthood Mental Health, Aged 3-30 Years in 2003, based on OLS Estimation**

|  | (1) | |
| --- | --- | --- |
| Dependent Variable | Depression  (CESD-20>16)) | |
| Variable | **Coef**  **(95% CI)** | ***P* value** |
| $\boldsymbol{Severity\times Young}$ | 0.067  (0.049-0.085) | <0.001 |
| Gender (male=1) | -0.005  (-0.026-0.015) | 0.614 |
| Years of education | -0.012  (-0.015- -0.008) | <0.001 |
| Married | -0.087  (-0.123- -0.051) | <0.001 |
| Employed | -0.063  (-0.095- -0.030) | <0.001 |
| Urban residence | 0.003  (-0.024-0.029) | 0.832 |
| City fixed effects | Yes | |
| Cohort fixed effects | Yes | |
| R2 | 0.068 | |
| Observations | 6289 | |

Abbreviations: Coef, coefficient; OLS, ordinary least squared.

The model controlled for sex, marital status, age, years of education, wealth index, urban residence, and survey month. Standard errors account for clustering at the city level.

**eTable 8: Associations Between Childhood SARS Severity on Adulthood Mental Health, Alternative Age Ranges in 2003, Based on Logistic Estimation**

|  | (1) | | (2) | | (3) | |
| --- | --- | --- | --- | --- | --- | --- |
| Dependent Variable | Depression  (CESD-20>16))  Sample age: 6-30 | | Depression  (CESD-20>16))  Sample age: 9-30 | | Depression  (CESD-20>16))  Sample age:9-18 | |
| Variable | **aOR**  **(95% CI)** | ***P* value** | **aOR**  **(95% CI)** | ***P* value** | **aOR**  **(95% CI)** | ***P* value** |
| $\boldsymbol{Severity\times Young}$ | 1.610  (1.376-1.885) | <0.001 | 1.778  (1.409-2.245) | <0.001 | 1.696  (1.381-2.082) | <0.001 |
| Gender (male=1) | 0.938  (0.818-1.075) | 0.357 | 0.950  (0.823-1.095) | 0.478 | 0.935  (0.760- 1.149) | 0.521 |
| Years of education | 0.933  (0.912-0.956) | <0.001 | 0.940  (0.918-0.963) | <0.001 | 0.953  (0.916-0.991) | 0.016 |
| Married | 0.586  (0.474-0.723) | <0.001 | 0.549  (0.438-0.688) | <0.001 | 0.602  (0.442-0.821) | 0.001 |
| Employed | 0.703  (0.577-0.856) | <0.001 | 0.694  (0.562-0.856) | 0.001 | 0.776  (0.585-1.030) | 0.079 |
| Urban residence | 1.013  (0.847-1.211) | 0.891 | 0.991  (0.829-1.186) | 0.925 | 0.852  (0.638-1.138) | 0.278 |
| City fixed effects | Yes | | Yes | | Yes | |
| Cohort fixed effects | Yes | | Yes | | Yes | |
| Pseudo R2 | 0.060 | | 0.065 | | 0.072 | |
| Observations | 5915 | | 5414 | | 2381 | |

Abbreviations: aOR, adjusted odds ratio; Coef, coefficient; OLS, ordinary least squared.

The model controlled for sex, marital status, age, years of education, wealth index, urban residence, and survey month. Standard errors account for clustering at the city level.

**eFigure 1: Permutation Tests on the Association of Childhood SARS Severity and Adulthood Depression, Aged 3-30 Years in 2003, Randomly Assigned SARS Severity Measure**

**
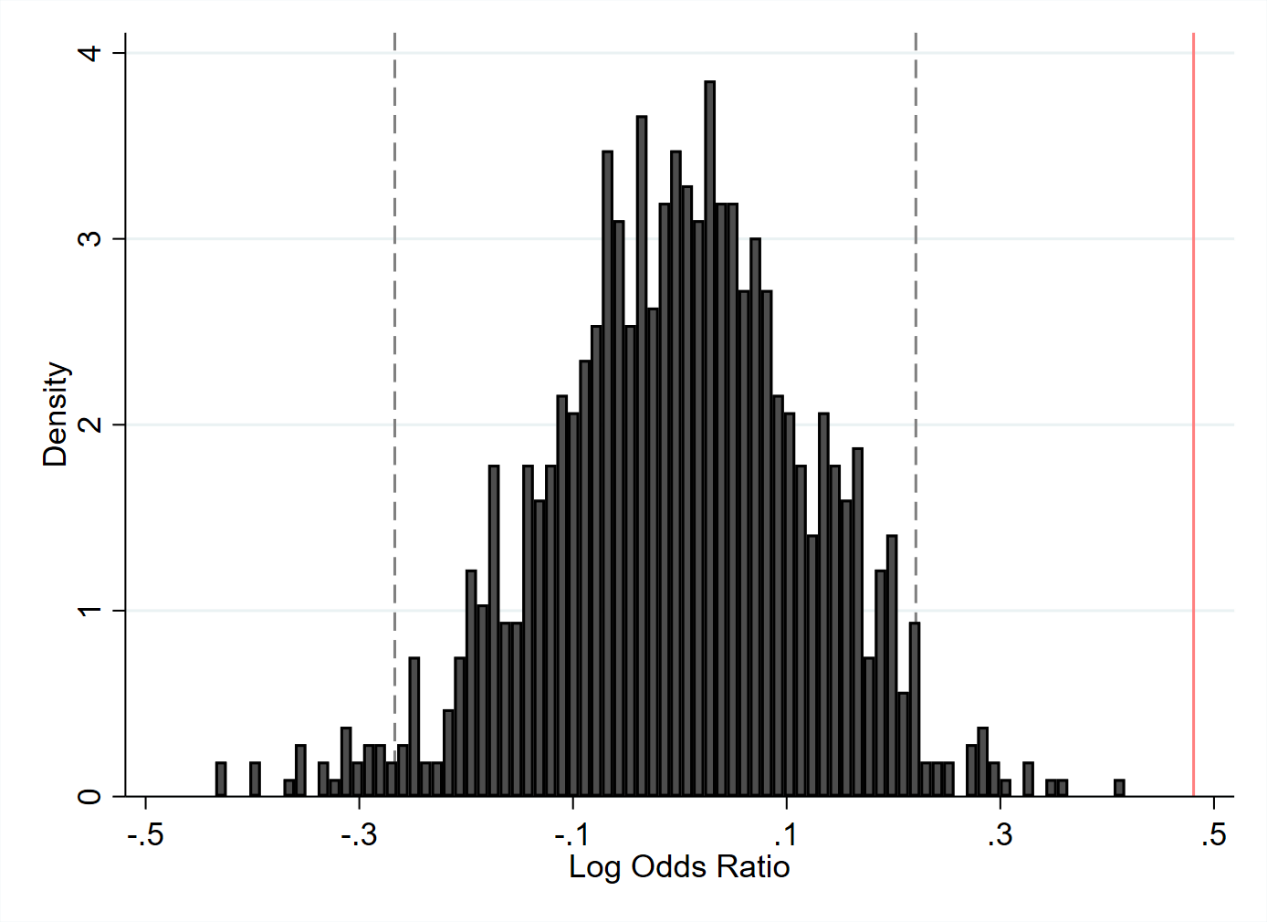
**

All models controlled for sex, marital status, age, years of education, wealth index, urban residence, and survey month. SARS severity measure was randomly assigned. Standard errors account for clustering at the city level.

**eFigure 2: Permutation Tests on the Association of Childhood SARS Severity and Adulthood Depression, Aged 3-30 Years in 2003, Randomly Assigned Individual’s Age in 2003**

**
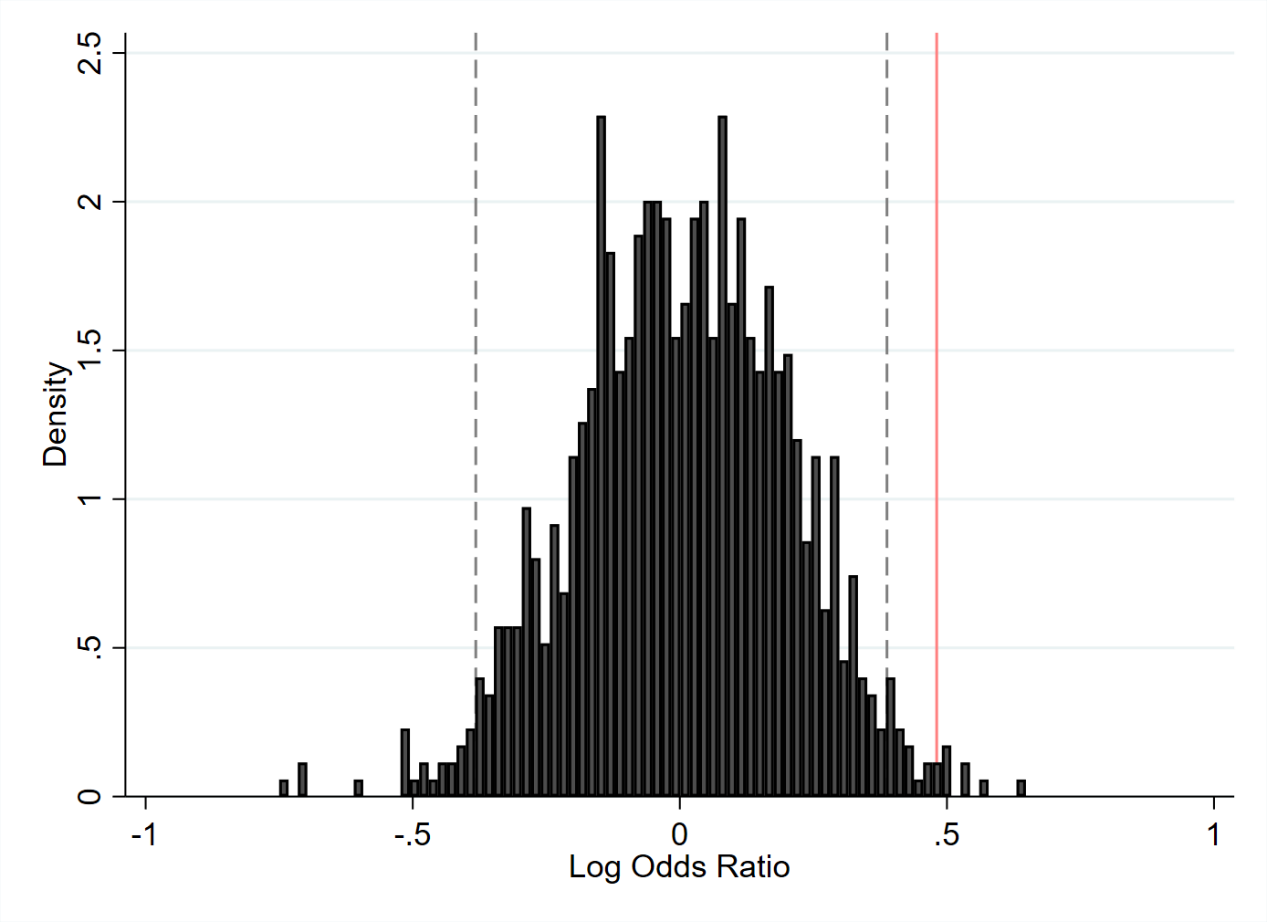
**

All models controlled for sex, marital status, age, years of education, wealth index, urban residence, and survey month. Individual’s age at year 2003 was randomly assigned. Standard errors account for clustering at the city level.

**eFigure 3: Distribution of Cumulative COVID-19 Cases in China**


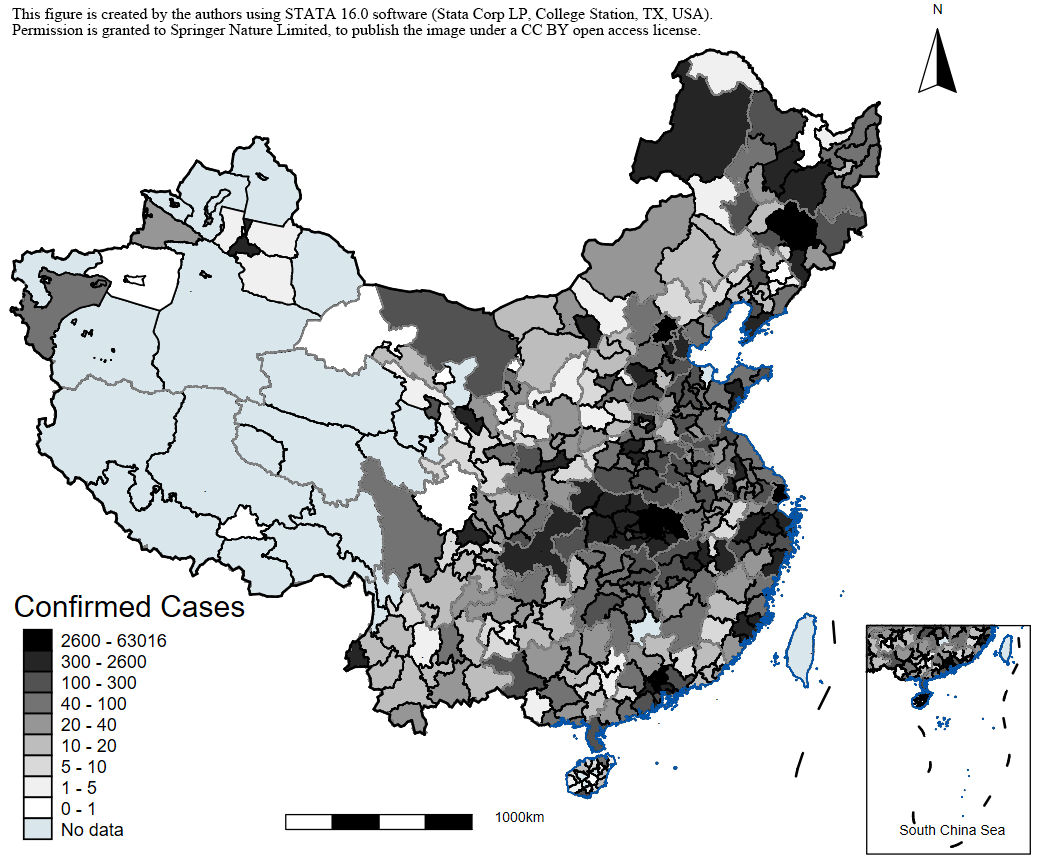


*Note:* This figure shows the geographical distribution of the city-level number of cumulative COVID-19 cases in a map of China from 2020.1.23 to 2022.6.1. Data from Hong Kong, Macau, and Taiwan are excluded.

This figure is created by the authors using STATA 16.0 software (Stata Corp, College Station, TX, USA). Permission is granted to Springer Nature Limited to publish the image under a CC BY open access license.
